# The Assessment of Visuospatial Skills and Verbal Fluency in the Diagnosis of Alzheimer’s Disease

**DOI:** 10.3389/fnagi.2021.737104

**Published:** 2022-01-20

**Authors:** Dalida Borbala Berente, Anita Kamondi, Andras Attila Horvath

**Affiliations:** ^1^School of Ph.D. Studies, Semmelweis University, Budapest, Hungary; ^2^Neurocognitive Research Center, National Institute of Mental Health, Neurology and Neurosurgery, Budapest, Hungary; ^3^Department of Neurology, Semmelweis University, Budapest, Hungary; ^4^Department of Anatomy Histology and Embryology, Semmelweis University, Budapest, Hungary

**Keywords:** Alzheimer’s disease, neuropsychology, verbal fluency, visuospatial abilities, cognitive domain

## Abstract

**Background:**

In the diagnosis of Alzheimer’s disease (AD), examining memory is predominant. Our aim was to analyze the potential role of various cognitive domains in the cognitive evaluation of AD.

**Methods:**

In total, 110 individuals with clinically defined AD and 45 healthy control participants underwent neuropsychological evaluation including Addenbrooke’s Cognitive Examination (ACE). Patients with AD were selected in three groups based on disease duration in years (Group 1: ≤2 years, *n* = 36; Group 2: 2–4 years, *n* = 44; Group 3: ≥4 years, *n* = 30). Covariance-weighted intergroup comparison was performed on the global cognitive score and subscores of cognitive domains. Spearman’s rho was applied to study the correlation between cognitive subscores and disease duration. The Wilcoxon signed-rank test was used for within-group analysis among ACE cognitive subscores.

**Results:**

Significant difference was found between ACE total scores among groups (χ^2^ = 119.1; *p* < 0.001) with a high negative correlation (*p* < 0.001; *r* = −0.643). With a longer disease duration, all the subscores of ACE significantly decreased (*p*-values < 0.001). The visuospatial score showed the strongest negative correlation with disease duration with a linear trajectory in decline (*r* = −0.85). In the early phase of cognitive decline, verbal fluency was the most impaired cognitive subdomain (normalized value = 0.64), and it was significantly reduced compared to all other subdomains (*p*-values < 0.05).

**Conclusion:**

We found that the impairment of verbal fluency is the most characteristic feature of early cognitive decline; therefore, it might have crucial importance in the early detection of AD. Based on our results, the visuospatial assessment might be an ideal marker to monitor the progression of cognitive decline in AD.

## Introduction

Currently, there are around 50 million patients worldwide living with major neurocognitive disorders. This number is expected to triple by 2050, placing a tremendous socioeconomic and medical burden on society. Alzheimer’s disease (AD) is the leading cause of the cognitive decline in older adults, accounting for two-thirds of dementia cases worldwide (Rasmussen and Langerman, 2019). AD is characterized by a gradual decline of cognitive function, affecting social and communication skills as well. The histopathological hallmarks of the disease are the presence of extracellular amyloid plaques and intracellular neurofibrillary tangles (Reitz et al., 2011). The initially affected neural structures are the hippocampus and the entorhinal cortex (Braak and Braak, 1991). These areas have a crucial role in episodic memory, spatial orientation, and visuospatial abilities.

The progression of the disease follows a pattern starting with mild cognitive impairment (MCI) as the prodromal phase of AD, which may appear years prior to the dementia diagnosis of a patient. In most patients, MCI is characterized by memory complaints (amnestic type MCI) (Mistridis et al., 2015). According to the current *Diagnostic and Statistical Manual of Mental Disorders*, 5th Edition (DSM-V) guideline, the short-term memory impairment becomes significant, and learning difficulties appear in mild AD (American Psychiatric Association, 2013). In moderate AD, other cognitive domains, including language difficulties and impaired orientation, are also involved. In severe AD, all cognitive domains are severely affected, and communication skills and self-reliance are lost (Förstl and Kurz, 1999).

Current diagnostic guidelines advise the evaluation of the medical history of a patient, clinical examination to test mental status as core tests and cerebrospinal fluid analysis, and neuroimaging using MRI or positron emission tomography (PET) as supportive diagnostic markers (Jack et al., 2018). The use of neuropsychological test batteries is also recommended [e.g., Montreal Cognitive Assessment (MoCA), Addenbrooke’s Cognitive Examination (ACE), and Alzheimer’s Disease Assessment Scale-Cognitive Subscale (ADAS-Cog)]. These tests focus mostly on assessing memory function and learning skills (i.e., the ratio of memory points/maximum score is 5/30 in MoCA, 35/100 in ACE, and 35/70 in ADAS-Cog), while the investigation of visuospatial abilities (i.e., the ratio of visuospatial points/maximum score is 4/30 in MoCA, 5/100 in ACE and 0/70 in ADAS-Cog) and verbal fluency (i.e., the ratio of verbal fluency points/maximum score is 1/30 in MoCA, 14/100 in ACE and 5/70 in ADAS-Cog) is relatively less detailed (Collie and Maruff, 2000). However, they might hold significant diagnostic and prognostic potential as well (Salimi et al., 2018) since they require the organized activation of large neural networks (Melrose et al., 2009; Quental et al., 2013; Ghanavati et al., 2019).

We hypothesized that, in AD, the severity of visuospatial- and verbal fluency performance decline is related to disease duration, as during the course of the neurodegenerative process more and more cortical areas involved in these functions become affected. Thus, our aim was to analyze the profile of cognitive impairment in patients with AD with various disease duration exploring multiple cognitive domains (memory, orientation, attention, verbal fluency, language, and visuospatial abilities) to assess their potential role in the early identification of AD and in the follow-up of the progression of cognitive decline.

## Materials and Methods

### Participants

In total, 110 participants (61 male, 49 female, and mean age = 73.1 ± 6.6 years) with clinically defined AD and 45 healthy control participants (16 male, 29 female, and mean age = 68.6 ± 7.40 years) were involved from the Department of Neurology at the National Institute of Mental Health, Neurology, and Neurosurgery (previously named as the National Institute of Clinical Neurosciences) in Budapest, Hungary. Informed written consent was obtained from each participant. The diagnosis of participants was given based on the guidelines of the National Institute on Aging and the Alzheimer’s Association (NIA-AA; McKhann et al., 2011). We sorted the participants with AD into three groups based on disease duration. Group 1 (*n* = 36) included participants with disease duration up to 2 years, group 2 (*n* = 44) with a disease duration of 2–4 years, and group 3 (*n* = 30) with a disease duration of 4 years or above. The healthy control individuals (Group 0; *n* = 45) had negative neurological status and intact cognitive performance based on neuropsychology. Disease duration was calculated from the date of clinical diagnosis of AD. The heteroanamnestic data were also collected from family members and caregivers. Patients with a history of cognitive symptoms more than 2 years prior to the diagnosis of AD were not included in this analysis. All methods were carried out in accordance with the relevant guidelines and regulations. All experimental protocols were approved by The Hungarian Medical Research Council (reference number of ethical approval: 024505/2015).

### Clinical Testing

The participants underwent detailed medical, neurological, physical examination, as well as routine blood checks including thyroid functions and vitamin B12 levels. All patients had structural brain MRI. The MRIs were analyzed with a visual inspection, and the medial temporal lobe atrophy (MTA) score was calculated. MTA = 1 shows that choroid fissure is slightly widened among the hippocampi, MTA = 2 shows a mild enlargement of temporal horn and mild loss of hippocampal height, MTA = 3 indicates moderate enlargement of temporal horn and moderate loss of hippocampal height, and MTA = 4 shows the marked enlargement of temporal horn and the loss of internal hippocampal structure (Duara et al., 2008). We determined all the known risk factors of cognitive decline as the exclusion criteria. Such risk factors included untreated vitamin B12 deficiency or hypothyroidism, liver disease, renal insufficiency, alcohol or substance abuse, psychoactive drugs influencing cognitive function except for anti-dementia medications, clinically significant brain lesions (white matter lesions, stroke), demyelinating conditions, head injury with loss of consciousness, hydrocephalus, schizophrenia, major depression, electroconvulsive therapy, HIV infection, syphilis, or prior central nervous system infections.

### Neuropsychology

All participants took part in neuropsychological evaluation. The assessments were conducted by trained neurologists or neuropsychologists. The language of the evaluation was Hungarian. We selected the Hungarian version of ACE (Stachó et al., 2003) to assess cognitive function. It is known for its high specificity and sensitivity in the diagnosis of cognitive disorders (Dudas et al., 2005). It tests six cognitive domains, namely, orientation, attention, memory, verbal fluency, language, and visuospatial abilities with a maximum score of 10, 8, 35, 14, 28, and 5, respectively, resulting in a maximum total score of 100. A total score of 83 as the cut-off score has an 82% sensitivity at age > 65 years (Mathuranath et al., 2000). Calculating the ratio of verbal fluency (V) and language (L) subscores/orientation (O) and delayed recall memory (M) subscores [VLOM ratio: (V + L)/(O + M)] enables differentiation between AD and frontotemporal dementia (FTD). The normal range of the VLOM ratio is between 2.2 and 3.2. A value higher than 3.2 indicates Alzheimer’s-type dementia, while a value lower than 2.2 demonstrates frontotemporal-type dementia. Visuospatial abilities are tested by asking the participant to copy two overlapping pentagons, to copy a cube, and to draw a clock face with the hands set at a specified time. Verbal fluency is analyzed with two tasks to examine categorical fluency (naming of animals) and phonemic fluency (listing words starting with the letter “m”). Furthermore, the Mini-Mental State Examination (MMSE) is incorporated in the ACE, enabling dementia severity assessment. Its total score ranges from 0 to 30, with higher scores indicating better cognitive performance. Patients with AD had MMSE < 25, while controls had >25.

Depression and anxiety may impair cognitive function (Kramer and Reifler, 1992; Seignourel et al., 2008). To reduce the influence of depression and anxiety on the data, we included the Beck Depression Inventory II (BDI-II) and Spielberger State and Trait Anxiety Inventory (STAI) in our test battery. A BDI-II score of less than 13 demonstrates minimal depression. Scores between 14 and 19 indicate mild depression, those between 20 and 28 refer to moderate depression, and a score of 29 or higher demonstrates severe depression. A low level of anxiety is indicated by a score of 45 or less for both state and trait anxiety. Participants with a BDI-II score of >13 or an STAI score of >45 were excluded from our analysis.

### Data Analysis

A recent study by de Boer et al. (2014) reported significant differences in MMSE total score and cognitive subdomain scores between three study groups of 125 patients with AD in total with various disease durations. Based on their results and our power calculations, the probability was equal or greater than 80% to find a significant (α = 0.05) difference between study groups in ACE total and cognitive subscores with a sample size of 150. Data distribution was tested using the Shapiro–Wilk test. To test for significant differences (for intergroup comparisons) in demographic variables (e.g., age and years of education), one-way ANOVA and Kruskal–Wallis tests were used as parametric and nonparametric tests, respectively, based on the distribution of data. Statistical significance level was set at *p* < 0.01 based on the Bonferroni correction due to multiple comparisons. Due to the nonparametric distribution of data, Spearman’s rho was used to study the correlation between disease duration (years) and cognitive function represented by the ACE total score. Between-group differences for ACE subscores were tested with covariance-weighted (age, sex, and disease onset) ANOVA and Kruskal–Wallis tests. Tukey’s test was applied for the *post hoc* analysis. Spearman’s correlation was applied for the connection of ACE subscores and disease duration. For within-group analysis including normalized ACE subscores, normalization was applied with the achieved score in each cognitive domain divided with a maximum possible score of the same cognitive domain (e.g., 7/28 in language cognitive domain resulted in 0.25). The normalized data were compared with the Wilcoxon signed-rank test due to the nonparametric distribution. IBM SPSS 20 software was used for statistical analysis.

## Results

### Demographic Data

Altogether 155 individuals (77 male: 49.7%; 78 female: 50.3%) participated in this study. The mean age of participants was 71.8 ± 7.1 years. The median duration of their education was 12 (12.0–17.0) years. Of the 155 participants, 45 were cognitively intact control individuals while 110 were diagnosed with clinically defined AD. On the brain MRI, patients showed the characteristic cortical atrophy (bifrontal-bitemporal atrophy with reduced hippocampi). All patients had MTA score ≥ 3.

Group 1 (*n* = 36; disease duration of no more than 2 years) included 23 male (63.89%) and 13 female (36.11%) participants with a mean age of 70.7 ± 7.4 years. In group 2 (*n* = 44; disease duration of 2–4 years), there were 25 male (56.8%) and 19 female (43.2%) participants. Their mean age was 74.1 ± 6.2 years. In group 3 (*n* = 30; disease duration longer than 4 years), 13 male (43.3%) and 17 female (56.7%) participants were selected, with a mean age of 74.6 ± 5.4 years. Group 0 included 45 control individuals (16 male (35.6%) and 29 female (64.4%) participants). Their mean age was 68.6 ± 7.4 years. We studied between-group differences in sex, age, age at disease onset, education level, disease duration, ACE total score, ACE subscores, and VLOM ratio (Table 1). Significant differences (*p* < 0.001) were reported in almost all parameters except sex and age at disease onset.

**TABLE 1:** Demographic and clinical data of participants.

Parameter	Total	Group 0	Group 1	Group 2	Group 3	*p*-value
Participants (*n*)	155	45	36	44	30	−
Female, *n* (%)	78 (50.3%)	29 (64.4%)	13 (36.11%)	19 (43.2%)	17 (56.7%)	0.936
Age (years) mean ± SD	71.8 ± 7.1	68.6 ± 7.4	70.7 ± 7.4	74.1 ± 6.2	74.6 ± 5.4	< 0.001
Age at disease onset (years) mean ± SD	70.2 ± 6.4	–	69.2 ± 7.3	71.1 ± 6.2	70.0 ± 5.6	0.43
Education (years) median ratio (IQ1–IQ3)	12.0 (12.0–17.0)	17.0 (12.0–17.0)	12.0 (12.0–16.5)	12.0 (12.0–17.0)	12.0 (10.0–15.0)	< 0.001
Disease duration (years) median ratio (IQ1–IQ3)	3.0 (2.0–4.0)	–	1.0 (1.0–2.0)	3.0 (3.0–3.0)	5.0 (4.0–5.0)	< 0.001
ACE total score median ratio (IQ1–IQ3)	72.0 (59.0–88.0)	94.0 (91.0–96.0)	72.0 (67.3–78.0)	66.5 (55.0–74.3)	50.0 (45.8–57.3)	< 0.001
VLOM median ratio (IQ1–IQ3)	3.3 (2.9–4.0)	2.6 (2.4–2.9)	3.5 (3.3–4.1)	3.5 (3.2–4.6)	3.6 (3.3–4.7)	< 0.001
MMSE median (IQ1–IQ3)	22.0 (17.0–28.0)	29.0 (28.0–29.0)	24.0 (21.3–25.0)	19.0 (16.0–21.0)	15.5 (12.8–18.0)	< 0.001
Orientation median ratio (IQ1–IQ3)	8.0 (7.0–10.0)	10.0 (10.0–10.0)	8.5 (8.0–10.0)	7.0 (6.0–8.0)	7.0 (5.0–8.0)	< 0.001
Attention median ratio (IQ1–IQ3)	7.0 (5.0–8.0)	8.0 (8.0–8.0)	6.0 (5.0–7.0)	6.0 (5.0–7.0)	5.0 (4.0–6.0)	< 0.001
Memory mean ± SD	21.0 ± 4.9	25.1 ± 1.8	21.9 ± 3.1	20.5 ± 4.4	14.2 ± 3.0	< 0.001
Verbal fluency median ratio (IQ1–IQ3)	9.0 (7.0–12.0)	13.0 (11.0–14.0)	9.0 (8.0–10.8)	8.5 (6.3–10.0)	7.0 (6.0–8.0)	< 0.001
Language median ratio (IQ1–IQ3)	23.0 (19.0–28.0)	28.0 (28.0–28.0)	24.0 (22.0–25.0)	20.0 (17.0–22.8)	17.5 (15.0–20.3)	< 0.001
Visuospatial abilities median ratio (IQ1–IQ3)	4.0 (4.0–5.0)	5.0 (5.0–5.0)	4.0 (3.3–5.0)	3.0 (2.0–3.0)	1.0 (0.75–2.0)	< 0.001

*Statistical tests applied were chi-square for sex, ANOVA for parametric statistics, and Kruskal–Wallis for nonparametric statistics. One-way ANOVA was used for between-group differences in memory. The Kruskal–Wallis test was used for between-group differences in orientation, attention verbal fluency, language, and visuospatial abilities. MMSE, Mini-Mental State Examination; IQ1–IQ3, interquartile range.*

### Relationship Between Addenbrooke’s Cognitive Examination Total Score and Disease Duration

Spearman’s rho showed a significant negative correlation between ACE total scores and disease duration (*p* < 0.001; *r* = −0.643). To support this finding, a one-way Kruskal–Wallis test was used, confirming significant group effect on the total ACE score (χ^2^ = 115.81; *p* < 0.001).

### Between-Group Differences Between Addenbrooke’s Cognitive Examination Subscores

**FIGURE 1:**
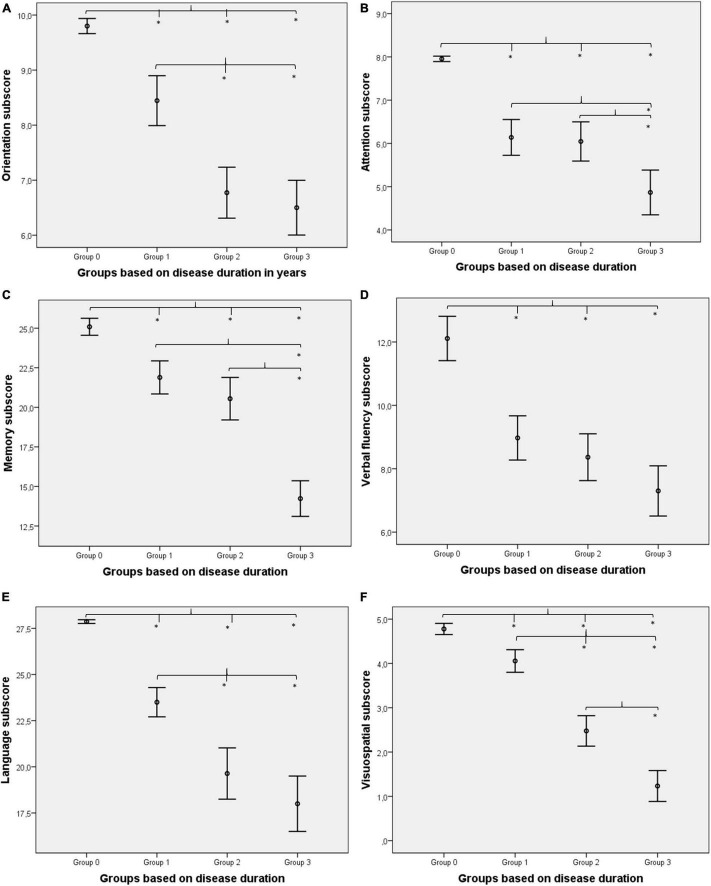
Between-group differences for cognitive subdomains. Orientation **(A)** was impaired in Alzheimer’s disease (AD) from the first 2 years of the disease compared to healthy controls (Group 1 vs. Group 0) and showed a gradual decline (rapid decline in the first 4 years and remains constant afterward). Attention **(B)** was impaired initially (Group 0 vs. Group 1), remained relatively preserved in the middle of the disease (Group 1 vs. Group 2), and deteriorated again in the later phase (Group 2 vs. Group 3). Memory **(C)** was also impaired from the first phase (Group 1 vs. Group 0) but did not show prominent changes in the first 4 years of the disease (Group 1 vs. Group 2), while a rapid decline was detectable in the later phase (Group 2 vs. Group 3). Verbal fluency **(D)** was highly damaged (largest difference between Group 0 and Group 1) in the first phase and did not decline further significantly. Language **(E)** was reduced initially (Group 1 vs. Group 0), and the linear decline was detectable in the first 4 years; however, changes were not so prominent at the end of the disease course (only Group 2 and Group 3 did not differ significantly). Visuospatial abilities **(F)** were reduced from the first phase also (Group 1 vs. Group 0), and linear deterioration was highlighted (all groups differed significantly). *Indicates significant differences (*p* < 0.01).

One-way ANOVA was used to test the between-group differences between the memory subscores (Table 1). Significant between-group differences were found for memory (*F* = 69.11; *p* < 0.001). The Kruskal–Wallis test was applied to study the between-group differences between the subscores of orientation, attention, verbal fluency, language, and visuospatial abilities (Table 1). Significant between-group differences were found for orientation (χ^2^ = 96.27; *p* < 0.001), attention (χ^2^ = 87.11; *p* < 0.001), verbal fluency (χ^2^ = 61.12; *p* < 0.001), language (χ^2^ = 100.38; *p* < 0.001), and visuospatial abilities (χ^2^ = 113.96; *p* < 0.001). Age, sex, and disease onset did not have a significant modifier effect on between-group differences (all *p*-values > 0.01). Tukey’s *post-hoc* analysis revealed that Group 1 differs from Group 2, Group 3, and Group 0 in orientation skills (all *p*-values < 0.001). Group 0 also differs from Group 2 and Group 3 in orientation skills (all *p*-values < 0.001); however, Group 2 and Group 3 are not significantly different (*p* = 0.779). In terms of attention subscore, Group 1, Group 3, and Group 0 all differ from each other significantly (all *p*-values < 0.001). Group 2 differs from Group 3 and Group 0 significantly (all *p*-values < 0.001). However, Group 1 and Group 2 do not differ significantly (*p* = 0.984). As for the memory subscore, Group 2, Group 3, and Group 0 all differ from each other significantly (all *p*-values < 0.001). Group 1 differs from Group 3 and Group 0 significantly (all *p*-values < 0.001). However, Group 1 and Group 2 do not differ significantly (*p* = 0.254). Regarding the subscore of verbal fluency, Group 0 differs from Group 1, Group 2, and Group 3 (all *p*-values < 0.001). However, Group 1 does not differ significantly from Group 2 and Group 3 (*p* = 0.629 and *p* = 0.017, respectively). Moreover, Group 2 does not differ significantly from Group 3 (*p* = 0.198). Concerning language subscore, Group 1, Group 3, and Group 0 all differ from each other significantly (all *p*-values < 0.001). Group 2 differs from Group 1 and Group 0 significantly (all *p*-values < 0.001). However, Group 2 and Group 3 do not differ significantly (*p* = 0.142). In terms of visuospatial subscore, all four groups differed significantly (all *p*-values ≤ 0.001; Figure 1). In comparison with normal controls (Group 0), verbal fluency showed the largest difference in the first phase of the disease (Group 1).

### Relationship Between Addenbrooke’s Cognitive Examination Subscores and Disease Duration

**FIGURE 2:**
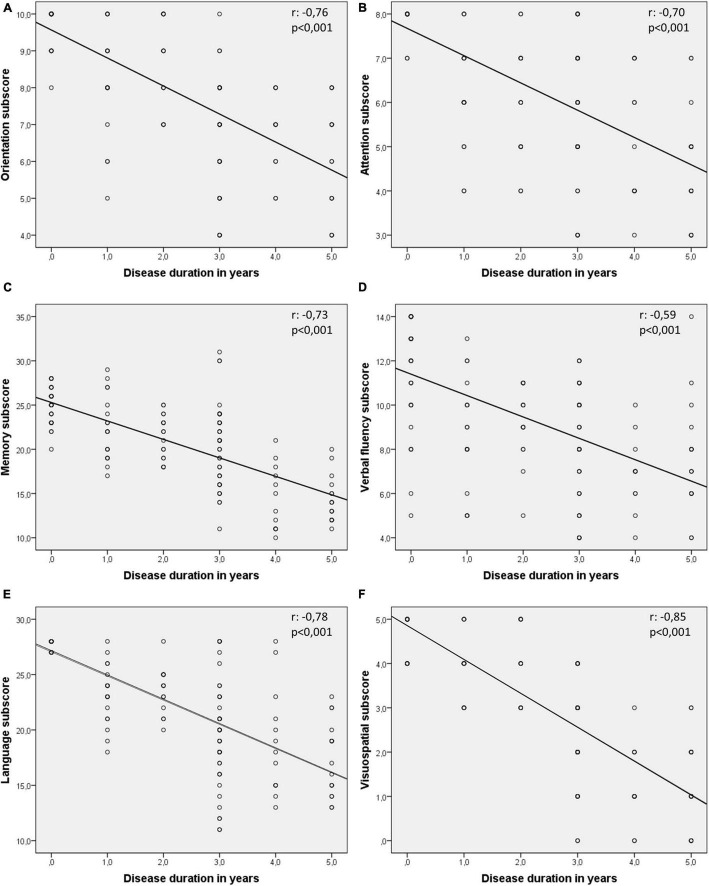
Correlation analysis between Addenbrooke’s Cognitive Examination (ACE) subscores and disease duration (in years) using Spearman’s rho. Significant negative correlation is present between all six subscores of orientation **(A)**, attention **(B)**, memory **(C)**, verbal fluency **(D)**, language **(E)**, and visuospatial **(F)** scores (all *p*-values < 0.05). Visuospatial abilities associate with the steepest *r* line.

Spearman’s rho was applied to test the relationship between all six ACE subscores and disease duration. Figure 2 demonstrates the scatter plots for subscores in relation to disease duration (Figure 2).

### Within-Group Differences Between Addenbrooke’s Cognitive Examination Subscores

**FIGURE 3:**
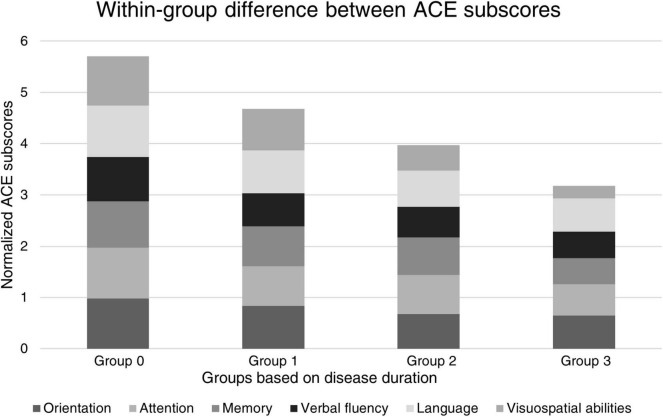
Within-group difference analysis for normalized ACE subscores. The contribution of verbal fluency in the cognitive maximum scores is the smallest in Group 1, suggesting prominent early impairment of this domain in the first phase of the disease. Noticeably, while the relative contribution of all cognitive domains did not change visually remarkably among the groups with various disease courses, visuospatial abilities showed a linear reduction in relative ratios.

We applied the Wilcoxon signed-rank test for within-group difference analysis between ACE subscores. Differences between the normalized subscores are shown in Figure 3 and Table 2.

**TABLE 2:** Normalized Addenbrooke’s Cognitive Examination (ACE) subscores for orientation, attention, memory, verbal fluency, language, and visuospatial abilities per group.

Cognitive subdomains	Descriptive statistics	Group 0	Group 1	Group 2	Group 3
Orientation	Mean	0.98	0.84	0.68	0.65
	SD	0.05	0.13	0.15	0.13
	Differences	O = A, O > M, O > VF, O < L, O > VS	O > A, O > M, O > VF, O = L, O = VS	O < A, O < M, O > VF, O = L, O > VS	O = A, O > M, O > VF, O = L, O > VS
Attention	Mean	0.99	0.77	0.76	0.61
	SD	0.03	0.15	0.19	0.17
	Differences	A > M, A > VF, A = L, A > VS	A = M, A > VF, A < L, A = VS	A = M A > VF, A > L, A > VS	A > M, A > VF, A = L, A > VS
Memory	Mean	0.90	0.78	0.73	0.51
	SD	0.06	0.11	0.16	0.11
	Differences	M = VF, M < L, M < VS	M > VF, M < L, M = VS	M > VF, M = L, M > VS	M = VF, M < L, M > VS
Verbal fluency	Mean	0.87	0.64	0.60	0.52
	SD	0.17	0.15	0.17	0.15
	Differences	VF < L, VF < VS	VF < L, VF < VS	VF < L, VF > VS	VF < L, VF > VS
Language	Mean	1.00	0.84	0.70	0.64
	SD	0.995	0.08	0.16	0.14
	Differences	L > VS	L = VS	L > VS	L > VS
Visuospatial abilities	Mean	0.96	0.81	0.50	0.25
	SD	0.08	0.15	0.23	0.19

*Normalization was performed by dividing the score of the participant in each cognitive domain by the highest score possible of the same domain (e.g., 5/10 in the orientation domain resulted in a normalized score of 0.5). Differences between the cognitive subscores were compared with the Wilcoxon signed-rank test. <, > indicate the statistically significant differences with the direction (p < 0.05), while = signals insignificant differences (p > 0.05). O, orientation; A, attention; M, memory; VF, verbal fluency; L, language; VS, visuospatial abilities.*

In Group 0, the normalized subscore of orientation was significantly higher than that of memory (*Z* = −4.083; *p* < 0.001), verbal fluency (*Z* = −3.95; *p* < 0.001), and visuospatial abilities (*Z* = −2.10; *p* = 0.036). However, the normalized subscore of orientation was significantly lower than that of language (*Z* = −2.32; *p* = 0.021). There was no significant difference between the normalized subscores of orientation and attention. The normalized subscore of attention is significantly higher than that of memory (*Z* = −5.40; *p* < 0.001), verbal fluency (*Z* = −4.60; *p* < 0.001), and visuospatial abilities (*Z* = −2.94; *p* = 0.003). There was no significant difference between the normalized subscores of attention and language. The normalized subscore of memory was significantly lower than that of language (*Z* = −5.52; *p* < 0.001) and visuospatial abilities (*Z* = −3.61; *p* < 0.001). There was no significant difference between the normalized subscores of memory and verbal fluency. The normalized subscore of verbal fluency was significantly lower than that of language (*Z* = −4.68; *p* < 0.001) and visuospatial abilities (*Z* = −3.75; *p* < 0.001). The normalized subscore of language was significantly higher than that of visuospatial abilities (*Z* = −2.82; *p* = 0.005).

In Group 1, the normalized subscore of orientation was significantly higher than that of attention (*Z* = −2.34; *p* = 0.019), memory (*Z* = −2.27; *p* = 0.023), and verbal fluency (*Z* = −4.79; *p* < 0.001). There was no significant difference between the normalized subscores of orientation, language, and visuospatial abilities. The normalized subscore of attention was significantly higher than that of verbal fluency (*Z* = −4.14; *p* < 0.001). However, the normalized subscore of attention was significantly lower than that of language (*Z* = −5.23; *p* < 0.001). There was no significant difference between the normalized subscores of attention, memory, and visuospatial abilities. The normalized subscore of memory was significantly higher than that of verbal fluency (*Z* = −4.41; *p* < 0.001). However, the normalized subscore of memory was significantly lower than that of language (*Z* = −5.23; *p* < 0.001). There was no significant difference between the normalized subscores of memory and visuospatial abilities. The normalized subscore of verbal fluency was significantly lower than that of language (*Z* = −5.23; *p* < 0.001) and visuospatial abilities (*Z* = −4.69; *p* < 0.001). There was no significant difference between the normalized subscores of language and visuospatial abilities.

In Group 2, the normalized subscore of orientation was significantly higher than that of verbal fluency (*Z* = −3.62; *p* < 0.001) and visuospatial abilities (*Z* = −4.38; *p* < 0.001). However, the normalized subscore of orientation was significantly lower than that of attention (*Z* = −3.23; *p* = 0.001) and memory (*Z* = −2.19; *p* = 0.029). There was no significant difference between the normalized subscores of orientation and language. The normalized subscore of attention was significantly higher than that of verbal fluency (*Z* = −5.47; *p* < 0.001), language (*Z* = −2.14; *p* = 0.032), and visuospatial abilities (*Z* = −5.24; *p* < 0.001). There was no significant difference between the normalized subscores of attention and memory. The normalized subscore of memory was significantly higher than that of verbal fluency (*Z* = −4.87; *p* < 0.001) and visuospatial abilities (*Z* = −5.25; *p* < 0.001). There was no significant difference between the normalized subscores of memory and language. The normalized subscore of verbal fluency was significantly higher than that of visuospatial abilities (*Z* = −3.31; *p* = 0.001). However, the normalized subscore of verbal fluency was significantly lower than that of language (*Z* = −3.55; *p* < 0.001). The normalized subscore of language was significantly higher than that of visuospatial abilities (*Z* = −4.07; *p* < 0.001).

In Group 3, the normalized subscore of orientation was significantly higher than that of memory (*Z* = −3.86; *p* < 0.001), verbal fluency (*Z* = −3.75; *p* < 0.001), and visuospatial abilities (*Z* = −4.73; *p* < 0.001). There was no significant difference between the normalized subscores of orientation, attention, and language. The normalized subscore of attention was significantly higher than that of memory (*Z* = −3.10; *p* = 0.002), verbal fluency (*Z* = −2.42; *p* = 0.016), and visuospatial abilities (*Z* = −4.74; *p* < 0.001). There was no significant difference between the normalized subscores of attention and language. The normalized subscore of memory was significantly higher than that of visuospatial abilities (*Z* = −4.46; *p* < 0.001). However, the normalized subscore of memory was significantly lower than that of language (*Z* = −4.32; *p* < 0.001). There was no significant difference between the normalized subscores of memory and verbal fluency. The normalized subscore of verbal fluency was significantly higher than that of visuospatial abilities (*Z* = −4.47; *p* < 0.001). However, the normalized subscore of verbal fluency was significantly lower than that of language (*Z* = −2.76; *p* = 0.006). The normalized subscore of language was significantly higher than that of visuospatial abilities (*Z* = −4.64; *p* < 0.001).

## Discussion

Our study involved 110 clinically defined patients with AD who were divided into three groups based on the length of disease duration. The control group (Group 0) consisted of 45 cognitively intact individuals. We found that verbal fluency is the most impaired cognitive domain in the first 2 years of the disease course, and its disturbance is comparable to the memory impairment in the early phase of AD. Furthermore, since visuospatial abilities showed the steepest reduction among the groups with various disease lengths, it might serve as an ideal method for monitoring disease progression.

Our analysis using correlation and between-group approaches showed that patients with longer disease duration have lower ACE global scores being in line with the current literature and confirming the fact that ACE indicates well the severity of AD (Hodges and Larner, 2017) and the global decline in cognition most frequently shows a linear pattern in AD (Suh et al., 2004; Wilkosz et al., 2010).

While a significant reduction in ACE subscores was present in a more advanced disease stage in the case of memory, verbal fluency, language, orientation, attention, and visuospatial abilities, the pattern of the impairment of various cognitive domains demonstrated prominent differences. Other studies also showed that the selective analysis of cognitive subdomains might reveal various trajectories of cognitive decline in AD (Wilkosz et al., 2010). Episodic memory impairment is the hallmark of AD; however, controversial results exist. Some reports suggest that declined episodic memory functions associate with the early phase of AD (Baudic et al., 2006; Sperling et al., 2010) while others suggest that prominent impairment occurs in the advanced phase of cognitive decline (Förstl and Kurz, 1999; de Boer et al., 2014). Our findings might reveal in-depth insight into the proposed problem. Our results show that memory is a highly affected cognitive domain already in the early course of the disease having a significantly lower normalized score (0.78) than any other subscores except attention (0.77) and verbal fluency (0.64). However, during the first 2–3 years after the diagnosis, the subsequent decrease of memory scores is not prominent (Groups 1 and 2 do not differ significantly in these subscores), suggesting that sequential memory testing might not be the ideal tool to sensitively detect the progression of the cognitive decline. However, memory functions show a rapid decline after 4 years of disease onset, supporting earlier data that demonstrated that memory impairment is predominantly evident in the later stages of AD (de Boer et al., 2014). This might suggest that while the global cognitive decline shows a continuously progressive course with the duration of the disease, episodic memory loss is becoming less pronounced while other domains contribute more in the linear global decline. From these data, we might conclude that testing memory independently is not appropriate to monitor disease progression or estimate the effect of disease-modifying interventions and drug trials in the mild and moderate phases of AD.

We also found that verbal fluency was even more severely compromised at the early stage of AD than memory (a normalized score of 0.78 for memory vs. a normalized score of 0.64 for verbal fluency). Other reports also highlighted that verbal fluency is impaired even in amnestic type MCI (Murphy et al., 2006) and in the preclinical phase or mild phase of AD (Clark et al., 2009). Ideal verbal fluency tests could not be developed for the routine screening of cognitive decline since there are controversial results: some studies propose that semantic (category) fluency might be an ideal tool for the early screening of dementia (Monsch et al., 1992; Pasquier et al., 1995; Gomez and White, 2006) while others demonstrated the superiority of phonemic (letter) fluency (Murphy et al., 2006). However, a meta-analysis of 153 studies with 15,990 participants proposed that semantic deficit is more prominent than phonemic (Henry et al., 2004). Based on our observations, it seems feasible that the development of novel and more focused diagnostic procedures on verbal fluency might be an important direction for the early screening of cognitive decline.

Our correlation analysis between disease duration and ACE subscores showed that patients with longer disease duration perform worse in all cognitive subdomain tests. The visuospatial score showed a remarkably strong negative correlation (larger than any other domains) with disease duration (*r* = −0.85), drawing special attention to this cognitive domain. Visuospatial skills are used to remember directions, addresses, and the layout of familiar places. Visuospatial abilities are tested by asking the patient to copy two diagrams, to draw a clock face with the hands set at a specified time, to count sets of dots, and to recognize four letters that are partially obscured. Although problems in visuospatial abilities are less well-characterized symptoms of AD compared to memory impairment (Salimi et al., 2018), visuospatial function monitoring could be ideal for assessing whether cognitive decline is progressive or not. Furthermore, it might be a useful cognitive test for the outcome measures of drug trials or lifestyle interventional studies.

There are limitations to our study. First, PET, cerebrospinal fluid analysis, or genetic testing was not applied in this experiment. Furthermore, the cognitive decline might appear years preceding the diagnosis of AD, so disease duration might vary among the examined patients. We involved patients with a short history of cognitive decline prior to the diagnosis of AD based on the reports of caregivers; however, the opinion of family members could be subjective. The strength of our study is the rigorous patient selection and the extensive application of different diagnostic methods.

## Conclusion

Alzheimer’s disease is the leading cause of dementia in older adults. However, only 16% of older adults receive regular cognitive evaluation (Alzheimer’s Association Report, 2019). Unfortunately, the estimated extent of missed or delayed diagnosis of AD is substantial (Bradford et al., 2009). The evaluation of the impairment of verbal fluency seems to have crucial diagnostic potential in the early identification of AD. Visuospatial abilities have been found to be impaired in AD even in preclinical stages and are considered to hold diagnostic potential (Hawkins and Sergio, 2014; Salimi et al., 2018). Furthermore, they might have a potential role in the assessment of progression of cognitive decline since they follow linear decline among the disease courses, so testing visuospatial skills might be ideal in the validation phase of drug trials.

## Data Availability Statement

The raw data supporting the conclusions of this article will be made available by the authors, without undue reservation.

## Ethics Statement

The studies involving human participants were reviewed and approved by the Hungarian Medical Research Council. The patients/participants provided their written informed consent to participate in this study.

## Author Contributions

DB was responsible for data management, the conduction of statistical analysis, and contributed to wrote the manuscript. AK was involved in the recruitment of patients, the design of the study protocol, and contributed to the correction of the manuscript. AH performed neuropsychological assessments, evaluated the results and concluded the major findings, and contributed to wrote the manuscript. All authors contributed to the article and approved the submitted version.

## Conflict of Interest

The authors declare that the research was conducted in the absence of any commercial or financial relationships that could be construed as a potential conflict of interest.

## Publisher’s Note

All claims expressed in this article are solely those of the authors and do not necessarily represent those of their affiliated organizations, or those of the publisher, the editors and the reviewers. Any product that may be evaluated in this article, or claim that may be made by its manufacturer, is not guaranteed or endorsed by the publisher.
